# Natural Antioxidant Activity of Commonly Consumed Plant Foods in India: Effect of Domestic Processing

**DOI:** 10.1155/2013/369479

**Published:** 2013-06-12

**Authors:** D. Sreeramulu, C. V. K. Reddy, Anitha Chauhan, N. Balakrishna, M. Raghunath

**Affiliations:** ^1^Endocrinology and Metabolism Division, National Institute of Nutrition, Jamai-Osmania Post Office, Tarnaka, Hyderabad, Andhra Pradesh 500007, India; ^2^Statistical Division, National Institute of Nutrition, Jamai-Osmania Post Office, Tarnaka, Hyderabad, Andhra Pradesh 500007, India

## Abstract

Phytochemicals protect against oxidative stress which in turn helps in maintaining the balance between oxidants and antioxidants. In recent times natural antioxidants are gaining considerable interest among nutritionists, food manufacturers, and consumers because of their perceived safety, potential therapeutic value, and long shelf life. Plant foods are known to protect against degenerative diseases and ageing due to their antioxidant activity (AOA) attributed to their high polyphenolic content (PC). Data on AOA and PC of Indian plant foods is scanty. Therefore we have determined the antioxidant activity in 107 commonly consumed Indian plant foods and assessed their relation to their PC. Antioxidant activity is presented as the range of values for each of the food groups. The foods studied had good amounts of PC and AOA although they belonged to different food groups. Interestingly, significant correlation was observed between AOA (DPPH and FRAP) and PC in most of the foods, corroborating the literature that polyphenols are potent antioxidants and that they may be important contributors to the AOA of the plant foods. We have also observed that common domestic methods of processing may not affect the PC and AOA of the foods studied in general. To the best of our knowledge, these are the first results of the kind in commonly consumed Indian plant foods.

## 1. Introduction

Reactive oxygen species (ROS) such as singlet oxygen, superoxide anion, hydroxyl radical, and hydrogen peroxide (H_2_O_2_) are often generated as byproducts of biological reactions or from exogenous factors [1]. These reactive species exert oxidative damage by reacting with nearly every molecule found in living cells including DNA [2]. Excess ROS, if not eliminated by antioxidant system, result in high levels of free radicals and lipid peroxides which underlie the pathogenesis of degenerative diseases like atherosclerosis, carcinogenesis, diabetes, cataract, ageing, and so forth [3]. 

Experimental and epidemiological evidence suggests a significant role of diet in the prevention of degenerative diseases [4]. Plant derived antioxidants, such as flavonoids and related phenolic compounds, have multiple biological effects, including antioxidant activity. Phytochemicals present in plant foods exert health beneficial effects, as they combat oxidative stress in the body by maintaining a balance between oxidants and antioxidants [5]. Although more than 8000 phytochemicals have been identified in plant foods, a large percentage remains to be identified. Further, data on the polyphenolic content antioxidant activity in Indian plant foods is scanty, and the effects of domestic processing on the AOA (antioxidant activity) in Indian plant foods are not reported yet [6].

Among plant foods, green leafy vegetables and grains are a rich source of antioxidants apart from energy, protein, and selected micronutrients in Indian diets [7]. Traditionally grains and GLVs have played a major role in providing nutrition particularly in the Indian Subcontinent and in other developing countries [8]. Since plant foods are often consumed in one or the other cooked forms, polyphenol and AOA intakes calculated on the basis of their content in raw foods are likely to be inaccurate. Therefore it was considered pertinent to study the effect of domestic processing on the natural antioxidant activity and phenolic content of commonly consumed plant foods rich in these activities. Hence the effect of domestic processing (cooking) was determined on antioxidant activity and polyphenol content in some commonly consumed green leafy vegetables (GLVs) and food grains. 

## 2. Sampling Procedures

The literature on antioxidant activity and phenolic content (PC) of plant foods is limited from India as well as other parts of the world. Available literature, mostly from other parts of the world, indicates that different researchers have adopted different sampling methods to get representative value of AOA and PC. Velioglu et al. [9] collected market samples and estimated AOA and PC in 200 mgs to 1 g of fruit, vegetable, and grain products. In another study, Al-Farsi et al. [10] took 1 g of sun-dried dates to estimate antioxidant parameters whereas Arcan and Yemenicioğlu [11] took about 20 g of fresh and dry nuts for the extraction. Sampling procedures followed in some Indian studies are as follows. Gupta and Prakash [12] used one gram of green leafy Vegetable for extraction whereas Nair et al. [13] have collected fresh food samples from local market, and five grams of cleaned food sample was taken to quantify PC and flavonoids in a few Indian plant foods. Keeping in view the differences in the sampling methods used and the quantities of samples extracted by different workers to analyse antioxidants in plant foods, it appears to be a good practice to take a higher quantity of food sample for the processing specially to get reproducible results and adopt ideal sampling practices for the quantification of AOA in food and herbal samples [6].

 Commonly consumed cereals, pulses, legumes, and GLVs analyzed in this study were chosen based on NNMB survey [14]. Samples were collected from market outlets located in three different locations of the twin cities of Hyderabad and Secunderabad, India. The market samples were pooled and analyzed in triplicates, and the results are presented as mean values on fresh weight basis. 

To determine the effect of different types of domestic processing of grains, edible portion of the sample was sorted out and divided into four aliquots of 25 grams each [11]. First portion was processed as such to know its natural (raw) antioxidant activity, while the 2nd, 3rd, and 4th portions of the sample were subjected to conventional, pressure, and microwave methods of cooking, respectively. We have mimicked consumer's habits of food procurement from market to household. In case of GLVs 10 g edible portions were taken and processed as above. 

## 3. Extraction Procedure

To determine the antioxidant activity in plant foods several solvent extraction procedures have been used by different researchers. There is no single satisfactory solvent extraction method suitable for the extraction of all classes of food antioxidants and phenolics. This probably is due to the differences in the chemical nature of antioxidants and phenolics, namely, simple to highly polymerized chemical substances present in plant foods. Oki et al. [15] used six different polar solvents to extract milled rice: n-hexane, diethyl ether, ethyl acetate, acetone, methanol and deionized water and found that the extracts with highly polar solvents like methanol, and deionized water shown the highest radical-scavenging activity. Al-Farsi et al. [10] used seven different solvents to extract sun-dried dates: water-phosphate buffer (40 : 60 ratio), methanol containing 0.1% formic acid (88 : 12 v/v), methanol/HCl (99.9 : 0.1% v/v), acetone containing 0.7% cyclodextrin, water (50 : 50 v/v), methanol/water (50 : 50 v/v), and water alone. They reported that most antioxidants present in dates were water-soluble (hydrophilic). On the other hand extraction with 50% methanol yielded the highest recovery of phenolics in the same study. This could be due to the solubility differences of phenolic acids in methanol and water. Therefore they used phosphate buffer for extracting antioxidant activity and 50% methanol to estimate total phenolic content in dates. 

Rochfort and Panozzo [7] used four different solvents to extract cereal grains: (i) acetone-water (80 : 10 v/v), (ii) ethanol-water (80 : 10 v/v), (iii) methanol-water (80 : 10 v/v), and (iv) water. Found that water and 80% methanol showed higher extraction than other solvents. In another study, Chidambara Murthy et al. [16] reported that methanol extracts of grape pomace protected the activities of hepatic enzymes and could thus be important in combating reactive oxygen species. In another study using *in vitro* models, Singh et al. [17] observed that methanol extracts of pomegranate peel and seeds had high antioxidant activity, and similar findings were reported by others [18]. Several workers used acidified 80% methanol extraction to assess antioxidant contents in plant foods, and the reasons for it could be that methanol extraction not only gives a higher yield but also gives high antioxidant activity as compared to that with other polar solvents. Hence we have used acidic, 80% methanol (with 0.1% HCl) for extraction of phenolics and AOA from foods in our studies. Methanol extracts were also used to know the effect of domestic cooking. Domestic cooking was done with normal tap water. 

Briefly, 10 grams of GLV or 25 grams of the grain sample was cooked in 100 mL of water for 10–15 minutes (in case of conventional cooking it took about 15 minutes; pressure cooking was done at 120°C for 10–12 minutes, and microwave cooking was done for 5–8 minutes, resp.). Cooking was done with the sample covered with lid except in conventional cooking. To estimate natural (raw) antioxidant content, the first portion of 10 or 25 g of the edible portion of the sample was ground in a domestic blender and extracted as such in 80% methanol containing 0.1% HCl, and final volumes of GLVs and grain samples were made to 50 and 100 mL extracts, respectively, with 80% methanol. 

## 4. Various Antioxidant Methods in Use

Determination of AOA in plant extracts is still an unresolved problem. It is not possible to evaluate multifunctional biological antioxidants in plant foods by a single antioxidant method. Hence, batteries of tests are used; about twenty different biochemical methods are in practice to asses AOA [19]. The exact comparison of the results obtained by different methods and their general interpretation may be practically impossible due to the variability of experimental conditions and differences in physicochemical properties of oxidizable substrates. Many other factors including colloidal properties of substrate, experimental conditions, reaction medium, oxidation state, and antioxidant localization in different phases may influence antioxidant activity. Among the different antioxidant parameters in use, ABTS (Trolox equivalent antioxidant assay TEAC/ABTS) and DPPH (2,20-Diphenyl-1-picryl hydrazyl) are widely used due to their simplicity, stability, accuracy, and reproducibility [20]. In a review on the AOA methods Huang et al. [21] suggested that FRAP (ferric reducing antioxidant power) and DPPH are the two most commonly accepted assays for the estimation of AOA in plant foods. In another study Ozgen et al. [22] evaluated the three most commonly used AOA methods and suggested that FRAP < DPPH < ABTS in fruits. A study carried out by Siddhuraju and Becker [20] suggested that DPPH < ABTS < FRAP showed better antioxidant and free radical-scavenging activities in processed cow pea and its seed extracts. Several new analytical approaches have suggested investigating antioxidant power of food extracts on the basis of their electron-donating ability. One such recently suggested assay is CAAP (chemiluminescence analysis of antioxidant power) which is a chemiluminescence based method. The rapid CAAP assay is said to be convenient to investigate the antioxidant power of herbal extracts. CAAP method showed positive correlation with FRAP (*r* = 0.959) [23]. Nevertheless, FRAP and DPPH assays are the most widely used methods. Since these assays are electron transfer based assays and often show excellent correlation with phenolic contents, and they are carried out in acidic conditions; pH values have an important effect on the reducing capacity of antioxidants. In acidic conditions reducing capacity tends to be suppressed due to protonation on antioxidant compounds, whereas in basic conditions proton dissociation of phenolic compounds would enhance the sample reducing capacity [24]. 

### 4.1. Phenolic Content

Soluble and hydrolysable phenolic contents (free phenols) were estimated as per the procedure described by Singh et al. [17] and Singleton and Rossi [25]. Values are expressed as Gallic acid equivalents. Colorimetric method was adopted in the present study, since sensitive chromatographic method in quantification of phenols is often limited to single class of phenolics and is often limited to low-molecular weight compounds that are available as standards. It is, therefore, necessary to use colorimetric assays such as the Folin-Ciocalteu assay which rely on the reducing ability of phenols to quantify the amount of total phenolics in a sample [26]. 

### 4.2. DPPH Radical-Scavenging Activity

 DPPH radical-scavenging activity was determined according to Aoshima et al. [27]. This method is based on the ability of the antioxidant to scavenge the DPPH cation radical. Briefly, to 100 *μ*L of sample extract or standard, 2.9 mL of DPPH reagent (0.1 mM in methanol) was added and vortexed vigorously. This was allowed to stand in dark for 30 min at room temperature, and the discoloration of DPPH was measured against a suitable blank at 517 nm. Percentage inhibition of the discoloration of DPPH by the sample extract was expressed as Trolox equivalents (mg/100 g).

### 4.3. FRAP Assay

 Ferric reducing antioxidant power (FRAP) was determined according to Benzie and Strain [28]. In the presence of TPTZ, the Fe^+2^-TPTZ complex exhibits blue color which is read at 593 nm. Briefly, 3.0 mL of working FRAP reagent was added to an appropriate volume/concentration of the sample extract, incubated for 6 min at room temperature, and the absorbance was measured at 593 nm against FeSO_4_ standard.

## 5. Over View 

Current life style is one of the major causes in the overproduction of free radicals and reactive oxygen species and decreasing physiological antioxidant capacity [29]. Food provides not only nutrients essential for life but also other bioactive compounds for health promotion and disease prevention. Epidemiological studies have consistently shown that regular consumption of plant foods is associated with reduced risk in developing chronic degenerative diseases and biological ageing [30]. Phytochemicals are the bioactive nonnutrient compounds present in plant foods which have been suggested to be responsible for their bioactivity linked to the reduced risk of major chronic diseases. It has indeed been estimated that a healthy diet could prevent approximately 30% of all cancers [31]. So far, published data from other parts of the world and India account only for a minor fraction of total polyphenols and AOA of plant foods. Therefore it was suggested to have food composition tables on antioxidant activity and polyphenolic content of commonly consumed plant foods from developing countries [32]. Hence we have attempted for the first time to get representative values of AOA in 107 commonly consumed plant foods in India. Purposive samples were purchased from three different local markets of the twin cities of Hyderabad and Secundrabad (India). They were analyzed separately and data presented on fresh weight basis, to mimic natural practice of consumption. PC and AOA were assessd in the methanol extracts of the foods by Folin-Ciocalteu method and DPPH/FRAP methods, respectively, and the results are expressed as Gallic acid and Trolox/FeSO_4_ equivalents, respectively. It has been observed in our studies that the foods studied had good amount of polyphenols and antioxidant activity, despite the fact that they belonged to different food groups. Also, a good correlation was observed between the natural AOA of the food and it's PC (Table 2) in many of the food groups studied. Part of natural antioxidant data was published by us as full length articles; hence range of values are given. Data on the effect of domestic processing on PC and AOA has been elaborated here since it is not yet published. 

## 6. Natural DPPH Activity in Group of Plant Foods

The range of DPPH activities in different food groups are presented in Table 1, and the values are expressed as mg/100 g on fresh weight basis. Among all the food groups analysed, the highest DPPH scavenging activity was observed in areca nut (28622 mg/100 g) while the activity was the least in carrots 11.06. The DPPH activity in cereals and millets ranged from 24–173 mg/100 g, with the highest activity being found in finger millet and the lowest in Semolina. The activity in legumes and pulses ranged 26–107 mg/100 g, with the highest in rajma and the lowest in roasted Bengal gram dhal. Among the nuts and oil seeds studied, the DPPH values ranged from 20–28622 mg/100, with areca nut showing the highest and coconut water having the least activities. Among the dry fruits DPPH activity ranged 271–1541 mg/100 g, with the highest activity being in walnuts and the lowest in piyal seeds. On the other hand among fresh fruits, the values ranged 32–891 mg/100 g, with the highest in guava and the lowest in watermelon. In green leafy vegetables the values ranged 21–1021 mg/100 g, with Curry leaves having the highest whereas spinach had the least activity. In roots and tubers category the DPPH activity ranged 11–125 mg/100 g, with the highest activity being found in red beet root and the lowest in carrot. Among the vegetables studied, the DPPH values ranged 12–466 mg/100 g. The highest activity was found in okra and the lowest was in ridge gourd. Due to scanty data available in the literature on DPPH activity in Indian plant foods it was not possible for us to compare these DPPH findings with the literature. 

## 7. Natural FRAP Activity in Group of Plant Foods

The range of FRAP activities in different food groups are presented in Table 1, and the values are expressed as mg/100 g on fresh weight basis. Among all the food groups analyzed, the highest FRAP activity was observed in areca nut 4220341 mg/100 g while the activity was the least in sunflower oil 36.10. Salient findings are as follows. Cereals and millets ranged 450–13093 mg/100 g, highest activity was found in finger millet, and the lowest was in Semolina. Among the dry fruits, activity ranged 1174–32416 mg/100 g, with the highest being in walnuts and the lowest in cashew nuts, whereas in fresh fruits, ABTS activity ranged 22–496 mg/100 g, with the highest in guava and the lowest was in pineapple. Some of these findings are in agreement with the literature values of fresh fruits [32]. The AOA determined by ABTS in fresh fruits and FRAP in dry fruits showed that both had reasonably good AOA. Interestingly dry fruits had higher activity than fresh fruits probably due to their low moisture content. It was pertinent to assess whether these observations made by two different methods in fresh and dry fruits could be validated by a common, third method. Therefore, we determined the AOA in fresh and dry fruits by the DPPH scavenging method, another most commonly used antioxidant biochemical parameter. FRAP activity in green leafy vegetables ranges 1380–27827 mg/100 g, mint leaves had highest activity, and the lowest was in spinach. Edible oils and sugars range 36–11674 mg/100 g, the highest activity found in jaggery and lowest in groundnut oil (unrefined). Among the nuts and oil seeds studied, FRAP ranges 220–4220341 mg/100 g; the areca nut showed the highest activity and lowest was in coconut water. In pulses and legumes FRAP ranged 1469–10362 mg/100 g, with the highest in rajma and the lowest in green gram dhal. The roots and tubers showed a wide range 256–6308 mg/100 g, and beet root had the highest and carrot the least. Among the vegetables studied, FRAP ranges 243–10510 mg/100 g. The highest activity was found in red cabbage and the lowest in pumpkin. Due to scanty data it was not possible for us to compare our FRAP finding with the literature. 

## 8. Natural Phenolic Content in Group of Plant Foods

The soluble total phenolic content (PC) data is presented in Table 1. Values are presented as mg Gallic acid equivalent/100 g on fresh weight basis. Among all the food groups analyzed, the highest PC was observed in areca nut 10841 GAE/100 g and was the least in coconut water 10.00. Coming to different food groups, in cereals/millets PC values ranged 47–373 mg/100 g; finger millets (Ragi) had the highest (373 mg/100 g), while milled rice had the lowest (47 mg/100 g). Dry fruits values range from 99 to 959 mg/100 g of which walnuts (959 mg/100 g) and piyal seeds (99 mg/100 g) had the highest and the lowest PC, respectively. PC of fresh fruits ranged from 26 to 374 mg/100 g, with the highest PC being in guava (374) and the least in watermelon (26 mg/100 g). PC of Papaya and sapota observed here are in agreement with reported data from other parts of the world [33], orange [34], pineapple [35], and apple [36]. Among the GLVs, PC was ranging 77–1077 mg/100 g; curry leaves have the highest (1077 mg/100 g) and the lowest was in spinach (77 mg/100 g). To compare our findings on natural phenolic contents of GLVs, as such there is very little or no published data available from India. However, Gupta and Prakash [12] analyzed phenolic content in 4 GLV samples, of which phenolic contents of fenugreek leaves values are comparable with our finding 158 versus 163 mg/100 g, whereas Amaranth and Curry leaves data is remarkably different from our findings 253 and 1077 mg/100 g, respectively, while the reported values are 150 and 387 mg/100 g. This variation could be due to the fact that they used tannic acid as standard whereas we used Gallic acid. However, it is not clear from Gupta and Prakash study [12] whether the data presented by them was on dry … or … on fresh weight basis was given. Coming to edible oils and sugars, the PC values ranged 0.72–336 mg/100 g. Jaggery had the highest PC (336 mg/100 g) while the lowest was in Vanaspati (0.72 mg/100 g). Nuts and oil seeds ranged from 10 to 10841 mg/100 g; areca nut had the highest phenolic content (10841 mg/100 g) and coconuts water the least (10 mg/100 g). Among the pulses and legumes, values ranged from 62–418 mg/100 g; black gram dhal had the highest (418 mg/100 g) while green gram dhal had the least (62 mg/100 g). Roots and tubers showed a wide range (22–169 mg/100 g), and beet root had the highest and carrot the least. Phenolic content of vegetables ranged from 27 to 339 mg/100 g, and red cabbage had the highest and ridge gourd the lowest. Very little published data is available on PC of Indian plant foods; some findings are in agreement with our data [37]. However, phenolic contents of plant foods can significantly vary due to various other factors, like plant genetics and cultivar, soil composition and growing conditions, maturity state and postharvest conditions, and so forth [38]. 

## 9. Correlation between PC, DPPH, and FRAP (in a Group of Natural Plant Foods)

Our observations on correlation between DPPH, FRAP, and PC of cereals, pulses, and legumes are in agreement with an earlier report [18] in that no significant correlation was observed between these two parameters among these food grains. Interestingly, no correlation was observed among PC, DPPH, and ABTS in wheat extracts [20]. The lack of correlation in cereal and legume grains could be due to the differences in the bound and free forms of phytochemicals present in them. It was observed that there was a possibility of underestimation of phenolic compounds in cereal/legume grains due to the differences in bound and free phenolics present in them. The bound phenolics contribute about 62% in rice to 85% in corn [5]. Another possibility could be due to the different responses of different phenolic compounds in different assay systems. Since the molecular antioxidant responses of phenolic compounds vary remarkably, depending on their chemical structure, their AOA does not necessarily correlate with the PC in grains [39]. 

However, both in dry and fresh fruits, there was a good correlation between PC and AOA, and our findings are in agreement with the available literature on the phenolic content of fresh and dry fruits [10]. However the discordance in phenolic content of different groups of foods studied could be due to varietal, seasonal, agronomical, and genomic differences, moisture content, method of extraction and standards used, and so forth [40]. Among GLVs, there was a good correlation among the PC and antioxidant parameters studied (Table 2). However, little information is available in the literature on the AOA and PC correlations in GLVs [12].

Although edible oils and sugars, belong to different food groups, there was a good correlation among their PC and AOA parameters in that the “*r*” value was 0.93 between both the AOA parameters and PC. Among nuts and oil seeds, a significant correlation was observed between AOA (both DPPH and FRAP) and PC. The “*r*” value between PC and AOA was 0.99, indicating the importance of PC to their AOA as assessed by these two methods. These findings are in agreement with earlier reports of this nature [10].

Correlations between the antioxidant activity and phenolic content of roots, tubers, and vegetables are given in Table 2. In general, there was a good correlation between the PC and AOA among the vegetables, roots, and tubers studied. A significant correlation (*P* < 0.01) was observed between PC and AOA both in roots and tubers (*r* values being 0.76 and 0.85, resp. with DPPH and FRAP) and other vegetables (*r* = 0.79 and 0.85 with DPPH and FRAP). These findings suggest that PC may be an important contributor to the AOA of roots, tubers, and vegetables; our observations are in agreement with the literature from other parts of the world [34]. 

## 10. Effect of Domestic Cooking on PC and AOA in Green Leafy Vegetables

 Plant foods are often consumed in one or the other processed forms. Therefore, it was considered pertinent to study the effect of common domestic processing (cooking) methods on the natural antioxidant activity and phenolic content of a few commonly consumed plant foods. Since oxidants and antioxidants have different chemical and physical characteristics, different types of cooking may bring different type of alterations in antioxidant activities of different foods. Further, if polyphenol intakes are calculated based on raw plant foods, the intake values computed may not be accurate. Hence effect of cooking was determined on phenolic content and antioxidant activity in commonly consumed green leafy vegetables (GLVs) and food grains.

Effect of cooking on PC and AOA of GLVs is presented in Tables 3–6. Phenolic content and antioxidant activity of foods cooked by different methods were compared with its natural (raw) activity from same portion of subsample. In general different cooking methods used in this study did not affect the AOA and phenolic contents in most of the GLVs. Percent change in the phenolic content (PC) or antioxidant activity (AOA) on cooking is given in parentheses with respective raw GLV value (Tables 3–6). Differences were considered significant at a *P* value at least <0.05.

 Out of the eleven GLVs studied, only two GLVs, namely, Ambat Chukka and Ponnaganti showed a small decrease of 10–20% in their PC content on cooking. While Gogu showed very little or no change on cooking. The other eight GLVs showed an increase in PC during different types of cooking (Table 3). Among them six GLVs, namely, Amaranth, Curry leaves, Fennel, Fenugreek, Purslane and Sorrel leaves showed an increase in PC, ranging 108−146% on cooking. Coriander, Mint, and spinach showed a significant increase in PC in the above cooking methods, and the increase was ranging 125–211% (Table 1). As such there is very little data of this kind reported in the literature. Kuti and Konuru [41] demonstrated in spinach leaves a similar increasing trend in PC on cooking. Contrary to this, Faller and Fialho [42] showed cooking loss in PC in vegetables. The possible explanation for the increasing or decreasing trends of phenolic contents during various cooking methods could be that the phenolics are stored in pectin or cellulose networks of plant foods and can be released during thermal processing. In turn individual phenolics may sometime increase because heat can break supramolecular structure which might make the phenolic compounds react better with the reagents [43]. 

Effect of cooking on DPPH scavenging activity is given in Table 4. The increase or decrease activity in different GLVs during different cooking methods was compared with its natural DPPH activity of the raw GLV. Our findings on changes in DPPH are in line with those in PC. In general, an increasing trend in DPPH activity on cooking was seen in most of the GLVs studied. Out of eleven GLVs, marginal effect of 1–10% was seen only in Ambat Chukka. Most of the GLVs showed an increasing trend in all the three methods of cooking. Among them Purslane and Ponnaganti showed 10–20% increase, whereas Amaranth and Mint showed 17–50% increase. Coriander and Curry leaves showed an increase of 38–133%. During conventional cooking, curry leaves showed little effect (<7%) while spinach showed an enormous increase of 221–381%. Remaining three GLVs namely, Fennel, Fenugreek, and Gogu leaves did not show any effect in conventional and pressure cooking but in microwave cooking alone showed about 31–36% increase (Table 4). This could be due to effect of high temperature as compared to the above two methods of heat treatment. Considering that no data of similar type is available from other parts of the world, we are unable to compare our findings with literature reports. Data available on other vegetables (not GLVs) reported a mixed trend, which is in agreement with our results. Indeed an increasing trend was observed in potatoes [44], while a decreasing trend was reported in other vegetables [42]. 

 Effect of cooking on FRAP is presented in Table 5. This biochemical indicator was chosen as the second most commonly used antioxidant biochemical parameter. Again, similar increasing trends were seen in FRAP activity on cooking. Most of the GLVs (nine out of eleven) showed an increase, ranging 119–181%. While Coriander and spinach leaves showed an enormous increase, two other GLVs, Ambat Chukka and Ponnaganti, showed a decrease (maximum of 10%). This type of complex trend during cooking requires further research [45]. However, the present data on natural antioxidant content in commonly consumed GLVs is the first data of its kind from India. Secondly our findings on the effect of different methods of cooking in above GLVs, most of them, show an increase in AOA; it could be due to better availability of bound phenolics. Correlation among the three biochemical parameters and effect of different methods of cooking were assessed next, and these correlations were compared by using rank correlations (Table 6). Phenolics versus antioxidant parameters in different cooking methods are highly correlated. Findings of this study suggest that although different cooking methods showed changes (highly significant in some cases) in the phenolic content and AOA of the GLVs, there was no effect of domestic cooking on the correlation between the PC and AOA. This observation confirms that PC may be important contributor to the AOA of GLVs both in raw and cooked forms. 

## 11. Effect of Domestic Cooking (Food Grains)

PC and AOA of the food grains (raw and cooked by different methods) are presented in Tables 7–9. The PC of raw whole green gram (with peel) was the highest (284 mg/100 g) followed by black rajma (146 mg/100 g). Green gram dhal without peel had the least phenolic content (41 mg/100 g) (Table 7). This difference in phenolic content of green gram whole and dhal could be due to the peel component, known to contribute high phenolic contents in grains. DPPH scavenging activity was the highest in black rajma (160 mg/100 g) followed by whole green gram (113 mg/100 g), and the lowest was in green gram dhal (without peel) (21 mg/100 g) (Table 8). FRAP content was the highest in black rajma followed by soya bean and the lowest was in green gram dhal. The FRAP values were 6852, 3778, and 1066 mg/100 g, respectively (Table 9).

Effect of different cooking methods on antioxidant activity of each food grain was compared with its AOA and phenolic contents of the raw sample. (Tables 7–9). Percentage change in the PC and antioxidant activity on cooking is given in parentheses in Tables 7–9. Overall, different cooking methods did not show any significant cooking losses but showed mixed results of increasing and/or decreasing trends (Tables 7–9), the changes being significant in most of the whole grains as compared to grains without seed coat.

Effects of cooking on PC are presented in Table 7. Nine out of 11 legumes samples showed the maximum of 20% increase or decrease in their PC during different types of domestic cooking. Interestingly, during conventional and pressure cooking, whole Bengal gram and rajma have shown 27 and 54% increase. Other studies showed similar effects on AOA in potatoes upon cooking [44] and in other vegetables [38]. The possible mechanism for the increase or decrease in AOA during various cooking methods could be that the phenolics were stored in pectin or cellulose networks of plant foods and were released during thermal processing [39].

DPPH scavenging activity in legumes cooked by different cooking methods also showed a mixed/inconsistent trend (Table 8). Nine out of eleven food grains studied showed less than 20% increase or decrease during cooking. It is however interesting that whole green gram (with peal) showed a higher increase in DPPH activity in all cooking methods studied, with the increase ranging 40–62% as compared to its content in the unprocessed form. Indeed some literature says that this type of complex trend on cooking is unexplainable and requires further research [45]. 

Effect of cooking on FRAP activity is given in Table 9. Findings are in line with DPPH, showing a mixed trend. Nine out of eleven legumes showed less than 20% variation in FRAP values. While whole green gram and dry green peas showed higher increase in FRAP ranging 41–102% in different methods of cooking; lentil and red gram dhal showed 34–73% increase *albeit* during pressure cooking only. It was however of interest that over all the percent increase or decrease found vis-à-vis their content in unprocessed food showed similar trend in different cooking methods in a given food grain. Such increasing or decreasing trends were reported in few vegetables from other parts of the world [46]. The possible explanation given for this type of finding was summarized by few workers as follows. Cooking could have resulted in liberation of high amounts of antioxidant compounds due to thermal destruction of cell wall and subcellular compartments [47, 48]. Another possibility might be the production of stronger radical-scavenging antioxidants by thermal or chemical reactions [49]. There can be a production of new nonnutritional antioxidants or formation of novel compounds such as Millard reaction products with antioxidant activity during cooking. However these findings are first of their kind in commonly consumed pulses and legumes.

Correlations among the PC and AOA were determined in the legumes in unprocessed as well as during the three different types of domestic cooking. For this purpose rank correlations were used, and the data is presented in Table 10. Correlations between PC and AOA were significant in different cooking methods, and they were comparable across the methods. Although different cooking methods showed changes (highly significant in some cases) in the phenolic content and AOA of the food grains, the finding that they did not affect the correlation between the PC and AOA suggests that PC may be important contributor to the AOA even in pulses and legumes, both in raw and cooked forms. 

## 12. Conclusions

 To the best of our knowledge, findings observed in this review are first of their kind from India, this review mainly dealt with two aspects, and natural antioxidant content of commonly consumed plant foods in India was assessed and correlated with its phenolic content. And the second aspect is assessing the effect of domestic cooking on PC and antioxidant activity for the first time from India in the most commonly consumed GLVs and grains. Our findings demonstrate that antioxidant contents did not get affected in most of the foods studied; on the other hand most of them shown a higher AOA in different method of domestical processing. This overview would be useful to researchers, nutritionists, and consumers to assess AOA and/or formulate antioxidant-rich therapeutic diets as well as commercial antioxidant-rich preparations from plant foods. In addition, they will be a valuable addition to the scanty knowledge on antioxidant activity of commonly consumed foods in India.

*Limitation of the Present Findings.* Purposive samples were collected from three local markets to provide first hand information on antioxidant activity of plant foods commonly consumed in India. Hence, findings cannot be considered as Indian plant foods data base.

## Figures and Tables

**Table 1:** Natural content of AOA and TPC.

S. no.	Group of foods	*n* (107)	Antioxidant content (mg/100 g)		PC (mg/100 g G A equ.)
			DPPH (Trol. equ.)	FRAP (FeSO_4 _equ.)	
1	Cereals and millets	9	24–173	450–13093	47–373
2	Dry fruits	10	271–1541	1174–32416	99–959
3	Edible oils and sugars	11	3–208	11–11674	0.72–336
4	Fresh fruits	14	32–891	22–496*	26–374
5	Green leafy vegetables	11	21–1020	1380–27827	77–1077
6	Nuts and oil seeds	12	20–28622	220–4220341	10–10841
7	Pulses and legumes	11	26–107	1469–10362	62–418
8	Roots and tubers	10	11–125	256–6308	22–169
9	Vegetables	19	12–466	243–10510	27–339

Values are expressed on fresh weight basis. *ABTS: range of values are given.

**Table 2:** Correlation between PC versus DPPH, FRAP.

S. no.	Group of Foods	*n* (107)	PC versus DPPH (*r*)	PC versus FRAP (*r*)	DPPH Versus FRAP (*r*)
1	Cereals and millets	9	0.45	0.91	0.84
2	Dry fruits	10	0.97	0.87*	0.81*
3	Edible oils and sugars	14	0.93	0.93	0.99
4	Fresh fruits	14	0.77	0.84*	0.94
5	Green leafy vegetables	11	0.94	0.95	0.96
6	Nuts and oil seeds	12	0.99	0.99	0.99
7	Pulses and legumes	11	0.16	0.44	0.78
8	Roots and tubers	10	0.76	0.85	0.97
9	Vegetables	19	0.79	0.85	0.75

*ABTS: correlations are in natural form.

**Table 3:** Effect of domestic processing on polyphenol content of commonly consumed green leafy vegetables.

Sl. no.	Common name	Botanical name	Phenolic content (mg/100 g Gallic acid Eq.)			
			Raw	Conventional	Pressure	Microwave
1	Amaranth	*Amaranthus gangeticus *	253.0^a^ (100)	275^b^ (108)	355^c^ (140)	312^d^ (123)
2	Ambat chukka	*Rumex vesicarius *	100.3 (100)	90 (89)	93 (92)	91 (91)
3	Coriander leaves	*Coriandrum sativum *	239.6^a^ (100)	417^b^ (174)	451^c^ (188)	506^d^ (211)
4	Curry leaves	*Murraya koenigii *	1077.0^a^ (100)	1434^b^ (133)	1184^c^ (109)	1377^d^ (127)
5	Fennel leaves	*Foeniculum vulgare *	251.3 (100)	268 (106)	265 (105)	312 (124)
6	Fenugreek leaves	*Trigonella foenum-graecum *	163.3^a^ (100)	180^a^ (110)	176^a^ (107)	220^b^ (134)
7	Purslane leaves	*Portulaca oleracea *	94.6^a^ (100)	128^b^ (135)	138^b^ (146)	128^b^ (135)
8	Sorrel leaves	*Hibiscus cannabinus *	191.3 (100)	194 (101)	211 (107)	213 (111)
9	Mint	*Mentha spicata *	440.3^a^ (100)	657^b^ (149)	796^c^ (180)	761^c^ (172)
10	Water amaranth	*Alternanthera sessilis *	136.3 (100)	122 (89)	110 (80)	123 (90)
11	Spinach	*Spinacia oleracea *	77.0^a^ (100)	96^b^ (125)	125^c^ (162)	117^c^ (152)

Mean values were compared (*n* = 3) by nonparametric Kruskal Wallis one way ANOVA. Differences in alphabets are significantly different at  *P* < 0.05. Percent gain or loss calculated when raw value taken as 100%. Percent recovery values are given in parentheses. Decimal points are not given due to higher numbers.

**Table 4:** Effect of domestic processing on DPPH activity of commonly consumed green leafy vegetables.

Sl. no.	Common name	Botanical name	DPPH (mg/100 g Trolox Eq.)			
			Raw	Conventional	Pressure	Microwave
1	Amaranth	*Amaranthus gangeticus *	405.6^a^ (100)	520^b^ (128)	527^b^ (129)	476^b^ (117)
2	Ambat chukka	*Rumex vesicarius *	85.3 (100)	87 (101)	83 (97)	94 (110)
3	Coriander leaves	*Coriandrum sativum *	471.0^a^ (100)	886^b^ (181)	948^b^ (201)	1100^c^ (233)
4	Curry leaves	*Murraya koenigii *	1020.6^a^ (100)	950^b^ (93)	1724^c^ (168)	1418^d^ (138)
5	Fennel leaves	*Foeniculum vulgare *	545.3 (100)	592 (108)	540 (99)	746 (136)
6	Fenugreek leaves	*Trigonella foenum-graecum *	144.3 (100)	142 (98)	127 (87)	193 (134)
7	Purslane leaves	*Portulaca oleracea *	138.3 (100)	162 (117)	165 (119)	151 (109)
8	Gogu	*Hibiscus cannabinus *	346.0 (100)	365 (105)	334 (96)	456 (131)
9	Mint	*Mentha spicata *	1368.6 (100)	2055 (150)	1856 (135)	2020 (147)
10	Ponnaganti	*Alternanthera sessilis *	173.0 (100)	172 (99)	203 (117)	198 (114)
11	Spinach	*Spinacia oleracea *	21.6^a^ (100)	69^b^ (321)	85^c^ (393)	104^d^ (481)

Mean values were compared (*n* = 3) by nonparametric Kruskal wallis one way ANOVA. Differences in alphabets are significantly different at  *P* < 0.05. Percent gain or loss calculated when raw value taken as 100%. Percent recovery values are given in parentheses. Decimal points are not given due to higher numbers.

**Table 5:** Effect of domestic processing on FRAP activity of commonly consumed green leafy vegetables.

Sl. no.	Common name	Botanical name	FRAP (mg/100 g FeSO_4_ Eq.)			
			Raw	Conventional	Pressure	Microwave
1	Amaranth	*Amaranthus gangeticus *	8237.6^a^ (100)	11370^b^ (138)	12102^b^ (146)	11786^b^ (143)
2	Ambat chukka	*Rumex vesicarius *	3511.6 (100)	3270 (93)	2946 (83)	3243 (92)
3	Coriander leaves	*Coriandrum sativum *	7125.6^a^ (100)	18636^b^ (261)	16123^c^ (226)	19802^d^ (277)
4	Curry leaves	*Murraya koenigii *	20275.0^a^ (100)	18533^b^ (91)	24213^c^ (119)	27392^d^ (135)
5	Fennel leaves	*Foeniculum vulgare *	9238.6^a^ (100)	10128^a^ (109)	9970^a^ (107)	13362^b^ (144)
6	Fenugreek leaves	*Trigonella foenum-graecum *	3409.6^a^ (100)	3919^b^ (114)	4799^c^ (140)	5429^d^ (159)
7	Purslane leaves	*Portulaca oleracea *	2863.3^a^ (100)	4327^b^ (151)	4800^c^ (167)	4030^b^ (140)
8	Gogu	*Hibiscus cannabinus *	5254.0 (100)	7274 (138)	6921 (131)	7107 (135)
9	Mint	*Mentha spicata *	27827.6^a^ (100)	42562^b^ (152)	48909^b,c^ (175)	50401^c^ (181)
10	Ponnaganti	*Alternanthera sessilis *	5068.3 (100)	4280 (84)	4837 (95)	4327 (85)
11	Spinach	*Spinacia oleracea *	1380.6^a^ (100)	3196^b^ (231)	3471^b^ (251)	3502^b^ (253)

Mean values were compared (*n* = 3) by nonparametric Kruskal wallis one way ANOVA. Differences in alphabets are significantly different at  *P* < 0.05. Percent gain or loss calculated when raw value taken as 100%. Percent recovery values are given in parentheses. Decimal points are not given due to higher numbers.

**Table 6:** Rank correlation between phenolic content versus DPPH and FRAP in different cooking methods of GLV.

TPC versus AOA	Raw	Traditional	Pressure	Microwave	Homogeneity
TPC versus DPPH	0.945	0.936	0.918	0.945	*χ* ^2^ = 0.23, *P* = 0.97
TPC versus FRAP	0.955	0.936	0.927	0.973	*χ* ^2^ = 1.23, *P* = 0.74
DPPH versus FRAP	0.964	0.973	0.991	0.991	*χ* ^2^ = 3.23, *P* = 0.36

All correlations are significant at  *P* < 0.001 (*n* = 11).

**Table 7:** Effect of domestic processing on polyphenol content of commonly consumed pulses and legumes in India.

Sl. No.	Common name	Botanical name	Phenolic content (mg/100 g Gallic acid Eq.)				*P* value
			Raw	Conventional	Pressure	Microwave	
1	Bengal gram dhal	*Cicer arietinum *	92.6 ± 5.5^a^ (100)	90.6 ± 6.5^a^ (98)	98.6 ± 4.0^a^ (106)	86.0 ± 5.5^a^ (93)	NS
2	Bengal gram dhal (roasted)	*Cicer arietinum *	116.3 ± 7.7^a^ (100)	105.6 ± 6.1^a^ (91)	108.6 ± 5.6^a^ (93)	102.0 ± 10.5^a^ (88)	NS
3	Bengal gram (whole grains)	*Cicer arietinum *	114.0 ± 10.4^a^ (100)	154.6 ± 7.0^b^ (136)	176.3 ± 4.5^c^ (154)	113.3 ± 6.0^d^ (99)	0.024
4	Black gram dhal (without peel)	*Phaseolus mungo Roxb *	69.3 ± 4.5^a^ (100)	58.6 ± 3.0^b^ (85)	60.0 ± 2.6^b^ (86)	51.3 ± 3.2^c^ (74)	0.022
5	Green gram dhal	*Phaseolus aureus Roxb *	41.3 ± 2.5^a^ (100)	43.6 ± 1.1^a^ (106)	43.0 ± 3.6^a^ (104)	34.0 ± 3.0^c^ (82)	NS
6	Green gram dhal (whole)	*Phaseollus aureus Roxb *	284.3 ± 6.5^a^ (100)	249.3 ± 3.0^b^ (88)	269.3 ± 4.5^c^ (95)	243.6 ± 4.0^b^ (86)	0.019
7	Lentil	*Lens esculenta *	64.3 ± 2.5^a^ (100)	64.6 ± 3.5^a^ (100)	59.0 ± 6.0^a^ (92)	56.0 ± 2.6^a^ (87)	NS
8	Peas green (dry)	*Pisum sativum *	82.3 ± 2.0^a^ (100)	84.0 ± 2.6^a^ (102)	103.3 ± 5.5^b^ (126)	75.6 ± 3.5^c^ (92)	0.024
9	Red gram dhal (without peel)	*Cajanus cajan *	70.0 ± 6.5^a^ (100)	83.6 ± 4.6^b^ (119)	81.6 ± 1.5^b^ (117)	74.0 ± 4.5^a^ (106)	0.035
10	Rajma (Black)	*Phaseolus Vulgaris *	146.6 ± 7.0^a^ (100)	186.0 ± 4.5^b^ (127)	195.6 ± 9.7^c^ (133)	159.3 ± 2.5^c^ (109)	0.020
11	Soya bean	*Glycine max* Merr.	81.6 ± 3.5^a^ (100)	82.0 ± 7.5^a^ (100)	98.3 ± 5.0^a^ (121)	94.3 ± 6.0^a^ (116)	NS

Pooled samples were analyzed in triplicates. Data is presented as mean ± SD. Mean values were compared by nonparametric Kruskal Wallies *H* test of one way ANOVA. Differences in alphabets are significantly different at  *P* < 0.05. Percent gain or loss calculated when raw value taken as 100%. Percent recovery values are given in parenthesis. Decimal points are not given due to higher numbers.

**Table 8:** Effect of domestic processing on DPPH activity of commonly consumed Pulses and Legumes in India.

Sl. no.	Common name	Botanical name	DPPH (mg/100g Trolox Eq.)				*P* value
			Raw	Conventional	Pressure	Microwave	
1	Bengal gram dhal	*Cicer arietinum *	42.6 ± 2.5^a^ (100)	43.3 ± 1.5^a^ (102)	43.6 ± 4.0^a^ (102)	40.0 ± 3.6^a^ (94)	NS
2	Bengal gram dhal (roasted)	*Cicer arietinum *	31.3 ± 2.5^a^ (100)	34.3 ± 3.7^a^ (110)	31.3 ± 3.5^a^ (100)	25.6 ± 2.5^a^ (82)	NS
3	Bengal gram (whole grains)	*Cicer arietinum *	68.6 ± 4.5^a^ (100)	100.0 ± 7.5^b^ (146)	95.3 ± 3.5^b^ (139)	60.3 ± 2.5^c^ (88)	0.022
4	Black gram dhal (with out peel)	*Phaseolus mungo Roxb *	35.0 ± 3.0^a^ (100)	29.0 ± 1.0^a^ (83)	30.0 ± 7.2^a^ (86)	24.6 ± .0^a^ (70)	NS
5	Green gram dhal	*Phaselus aureus Roxb *	21.3 ± 4.5^a^ (100)	19.3 ± 4.5^a^ (91)	17.6 ± 3.5^a^ (83)	18.6 ± 3.6^a^ (87)	NS
6	Green gram dhal (whole)	*Phaseolus aureus Roxb *	113.6 ± 9.2^a^ (100)	184.3 ± 9.0^b^ (162)	159.3 ± 13.7^c^ (140)	171.3 ± 9.0^b,c ^ (151)	0.027
7	Lentil	*Lens esculenta *	35.6 ± 3.5^a^ (100)	38.0 ± 4.0^a^ (107)	35.3 ± 3.7^a^ (99)	36.6 ± 6.5^a^ (103)	NS
8	Peas green (dry)	*Pisum sativum *	51.0 ± 3.0^a^ (100)	55.3 ± 3.0^a^ (108)	56.0 ± 4.0^a^ (110)	42.0 ± 5.5^b^ (82)	0.040
9	Red gram dhal (without peel)	*Cajanus cajan *	42.0 ± 4.0^a^ (100)	49.3 ± 7.5^a^ (117)	56.3 ± 4.7^a^ (134)	42.0 ± 4.0^a^ (100)	NS
10	Rajma (Black)	*Phaseolus Vulgaris *	160.0 ± 8.1^a^ (100)	182.3 ± 4.5^a^ (114)	170.3 ± 6.0^a^ (106)	174.0 ± 9.5^a^ (109)	NS
11	Soya been	*Glycine max* Merr.	75.6 ± 7.5^a^ (100)	61.3 ± 2.3^b^ (81)	59.3 ± 4.1^c^ (78)	71.6 ± 2.0^a^ (95)	0.023

Pooled samples were analysed in triplicates. Data is presented as mean ± SD. Mean values were compared by non-parametric Kruskal Wallies *H* test of one way ANOVA. Differences in alphabets are significantly different at  *P* < 0.05. Percent gain or loss calculated when raw value taken as 100%. Percent recovery values are given in parenthesis. Decimal points are not given due to higher numbers.

**Table 9:** Effect of domestic processing on FRAP activity of commonly consumed Pulses and Legumes in India.

S. no.	Common name	Botanical name	FRAP (mg/100 g FeSO_4_ Eq)				*P* Value
			Raw	Conventional	Pressure	Microwave	
1	Bengal gram dhal	*Cicer arietinum *	1679 ± 53.2^a^ (100)	1909 ± 64.7^a^ (114)	1968 ± 44.1^a^ (117)	1973 ± 46.6^a^ (118)	NS
2	Bengal gram dhal (roasted)	*Cicer arietinum *	1466 ± 125.2^a^ (100)	1711 ± 109.5^a^ (117)	1359 ± 114.5^a^ (93)	1367 ± 103.5^a^ (93)	NS
3	Bengal gram (whole grains)	*Cicer arietinum *	2283 ± 132.8^a^ (100)	2560 ± 131.0^b^ (112)	2676 ± 170.0^b^ (117)	2177 ± 102.1^a^ (95)	0.033
4	Black gram dhal (without peel)	*Phaseolus mungo Roxb *	1515 ± 41.4^a^ (100)	1420 ± 80.1^a^ (94)	1470 ± 46.5^a^ (97)	1265 ± 47.8^a^ (83)	NS
5	Green gram dhal	*Phaseolus aureus Roxb *	1066 ± 128.6^a^ (100)	1371 ± 58.3^a^ (128)	1042 ± 99.8^a^ (98)	938 ± 85.7^a^ (88)	NS
6	Green gram dhal (whole)	*Phaseolus aureus Roxb *	3098 ± 22.4^a^ (100)	5490 ± 101.0^b^ (177)	5785 ± 184.6^c^ (187)	5505 ± 81.1^b^ (178)	0.025
7	Lentil	*Lens esculenta *	1534 ± 54.0^a^ (100)	1652 ± 121.0^a^ (108)	2058 ± 109.0^a^ (134)	1625 ± 107.9^a^ (105)	NS
8	Peas green (dry)	*Pisum sativum *	1846 ± 80.8^a^ (100)	3027 ± 93.7^a^ (164)	3734 ± 71.0^b^ (202)	2609 ± 64.5^c^ (141)	0.016
9	Red gram dhal (without peel)	*Cajanus cajan *	2446 ± 84.9^a^ (100)	3133 ± 81.6^b^ (128)	4251 ± 106.6^c^ (173)	2646 ± 84.8^b^ (108)	0.016
10	Rajma (Black)	*Phaseolus Vulgaris *	6852 ± 66.4^a^ (100)	6809 ± 125.2^a^ (99)	7171 ± 81.4^b^ (105)	7915 ± 130.5^c^ (115)	0.025
11	Soya been	*Glycine max* Merr.	3778 ± 162.5^a^ (100)	3504 ± 128.0^a^ (93)	3714 ± 125.5^a^ (98)	3502 ± 149.0^a^ (93)	NS

Pooled samples were analysed in triplicates. Data is presented as mean ± SD. Mean values were compared by nonparametric Kruskal Wallies *H* test of one way ANOVA. Differences in alphabets are significantly different at  *P* < 0.05. Percent gain or loss calculated when raw value taken as 100%. Percent recovery values are given in parenthesis. Decimal points are not given due to higher numbers.

**Table 10:** Rank correlation between phenolic content and AOA (DPPH and FRAP) in raw and cooked pulses and legumes.

TPC versus AOA	Raw	Traditional	Pressure	Microwave	Homogeneity
TPC versus DPPH	0.689	0.801	0.793	0.780	*χ* ^2^ = 1.23, *P* = 0.746
TPC versus FRAP	0.573	0.701	0.619	0.706	*χ* ^2^ = 1.12, *P* = 0.772
DPPH versus FRAP	0.918	0.909	0.895	0.916	*χ* ^2^ = 0.31, *P* = 0.959

All correlations are significant at  *P* < 0.01  (*n* = 11), and correlations are comparable across the methods. Between the methods, all the parameters are significantly correlated (TPC versus DPPH, TPC versus FRAP, and DPPH versus FRAP).

## Abbreviations

AOA: Antioxidant activity
DPPH: 2,2′-Diphenyl-1-picryl hydrazyl
FRAP: Ferric reducing antioxidant power
GLV: Green leafy vegetables
TPTZ: 2,4,6-Tripyridyl-s-triazine
PC: Phenolic content.
